# Obesity-Specific Considerations for Assessing Gait with Inertial Measurement Unit-Based vs. Optokinetic Motion Capture

**DOI:** 10.3390/s24041232

**Published:** 2024-02-16

**Authors:** Julie Rekant, Scott Rothenberger, April Chambers

**Affiliations:** 1 Department of Bioengineering, University of Pittsburgh, Pittsburgh, PA 15213, USA; 2 Department of Medicine, University of Pittsburgh, Pittsburgh, PA 15213, USA; rothenberger@pitt.edu; 3 Department of Health and Human Development, University of Pittsburgh, Pittsburgh, PA 15213, USA; ajchambers@pitt.edu

**Keywords:** body mass index, gait biomechanics, accelerometry

## Abstract

Adults with obesity experience high rates of disability and rapid functional decline. Identifying movement dysfunction early can direct intervention and disrupt disability development; however, subtle changes in movement are difficult to detect with the naked eye. This study evaluated how a portable, inertial measurement unit (IMU)-based motion capture system compares to a laboratory-based optokinetic motion capture (OMC) system for evaluating gait kinematics in adults with obesity. Ten adults with obesity performed overground walking while equipped with the OMC and IMU systems. Fifteen gait cycles for each participant were extracted for the 150 total cycles analyzed. Kinematics were compared between OMC and IMU across the gait cycles (coefficient of multiple correlations), at clinically significant time points (interclass correlations), and over clinically relevant ranges (Bland–Altman plots). Sagittal plane kinematics were most similar between systems, especially at the knee. Sagittal plane joint angles at clinically meaningful timepoints were poorly associated except for ankle dorsiflexion at heel strike (ρ = 0.38) and minimum angle (ρ = 0.83). All motions except for ankle dorsiflexion and hip abduction had >5° difference between systems across the range of angles measured. While IMU-based motion capture shows promise for detecting subtle gait changes in adults with obesity, more work is needed before this method can replace traditional OMC. Future work should explore standardization procedures to improve consistency of IMU motion capture performance.

## 1. Introduction

More than 40% of adults in the United States have obesity [1]. Adults with obesity are known to walk differently than their normal-weight counterparts [2,3] and are at increased risk for mobility disability [4,5] and rapid functional decline following disability onset [6]. Early identification of mobility changes is important to direct interventions which can prevent further functional declines and help aging adults maintain functional independence. Gait analysis is an important tool to identify discreet but meaningful aberrancies in functional movement contributing to dysmobility [7,8]. Being able to identify these aberrancies early in adults with obesity is particularly important as those with a higher body mass accumulate greater loads over the 4000–10,000 daily steps most adults take [9], stressing vulnerable physiological structures and creating more pain than in those with lower body weights.

Leveraging technology to objectively analyze gait allows for the identification of subtle aberrancies that might be otherwise missed with standard visual observation [10]. Objective gait analysis is traditionally carried out in laboratory settings with expensive, computationally intensive optokinetic motion capture (OMC) systems. Fixed cameras track the location of reflective markers placed on anatomical landmarks, and then, reflective marker positions are imported into a computer model and joint center locations, and angles are calculated.

Portable, more affordable inertial measurement unit (IMU)-based motion capture systems pose a viable alternative to OMC and can be easily deployed in clinical settings. IMU systems utilize miniaturized wireless sensors strapped to body segments to determine joint angles and spatiotemporal characteristics of gait through laptop software [11,12,13]. IMU-based motion capture systems have been shown to be valid for gait assessment in normal weight populations when specific computational adjustments are implemented [14,15,16]. However, IMU-based motion capture has not been compared to OMC in adults with obesity, a group of adults particularly vulnerable to functional decline.

Further, previous research identified that biomechanical models for normal-weight adults are inaccurate and unreliable for describing mechanics of adults with obesity [17,18]. As a result, obesity-specific marker sets incorporating additional reflective and digitized markers around the pelvis have been established to more accurately measure gait biomechanics with OMC in this population [17]. Unfortunately, this adds complexity to an already computationally intensive process, further prohibiting clinical translation. Therefore, the goal of this analysis is to evaluate the validity of a portable, clinically implementable IMU-based motion capture system against the reference standard OMC for assessing kinematics of gait in adults with obesity. A better understanding of how this technology performs for adults with obesity will aid in its responsible translation into clinical settings for biomechanical assessments, leading to earlier identification of movement aberrancies and promoting earlier intervention to support functional independence with aging in this population.

## 2. Materials and Methods

### 2.1. Study Population

This study included middle-aged adults (40–64 years old) with obesity (body mass index (BMI): 30–45 kg/m^2^) and without a history of balance or dizziness problems, taking medications affecting balance, or orthopedic injuries within the prior three years. Middle aged adults with obesity were chosen for this analysis as this has been identified as a time of disability development, presenting an opportunity to detect subtle, early changes in dysmobility that are indicative of future overt disability development [10]. All participants self-reported being “overall healthy”. Participants completed informed consent prior to participation in study procedures. Visits were conducted at the University of Pittsburgh’s Human Movement and Balance Laboratory using a study protocol approved by the University of Pittsburgh’s Institutional Review Board.

### 2.2. Procedures

**Figure 1:**
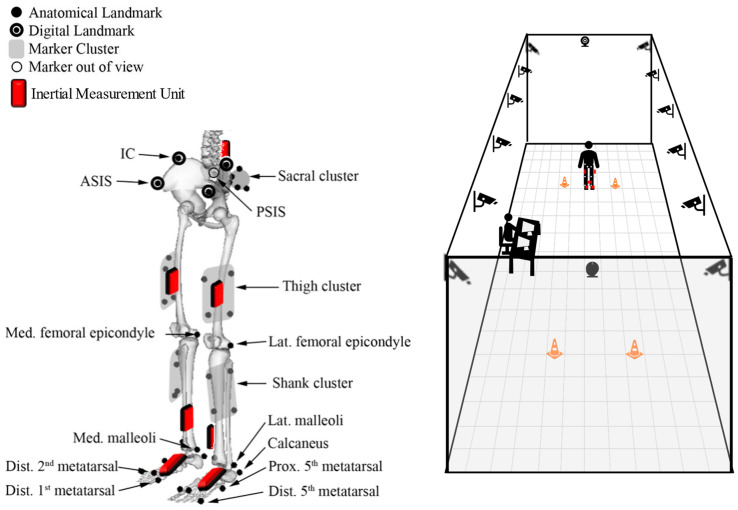
Schematic of OMC marker and IMU sensor locations (**left**). Figure adapted from Lerner et al., 2014, with permission from the author [17]. Schematic of data collection room and motion capture camera locations (**right**).

For this population-specific validation, an OMC obesity-specific marker set [17] was compared to the standard Noraxon MyoMotion IMU set recommended by manufacturers (Noraxon USA, Scottsdale, AZ, USA). IMUs were placed in accordance with previously described methodology (Figure 1) [14]. A single, static calibration captured during standing in the anatomical position was applied to all Noraxon data collection files prior to each walking bout. Participants walked at self-selected speeds along a 30-foot level walkway. The walkway was instrumented with 14 Vicon motion capture cameras (Vicon Motion Systems Ltd., Centennial, CO, USA) and with simultaneous collection from the Noraxon MyoMotion module (Figure 1). IMU data were collected at 100 Hz, and OMC data were collected at 120 Hz.

### 2.3. Data Analysis

Data were temporally synced with a trigger during data collection; a square wave outputted from the trigger initiated simultaneous data collection in both the OMC and IMU software systems. Initiation of the foot motion was visually examined during post-processing to verify that data were properly synced. The Noraxon MyoMotion system automatically calculates sagittal plane angles at the knee and sagittal, frontal, and transverse plane angles at the ankle and hip using proprietary software (Noraxon USA, Scottsdale, AZ, USA). A custom MATLAB code (Version R2018a, MathWorks Inc., Natick, MA, USA) calculated these same seven kinematics from OMC data. International Society of Biomechanics (ISB) convention was used [19,20]. Prior to analysis, OMC data were filtered with a 4th order 10 Hz low-pass Butterworth filter [21]. Dominant leg heel strikes were used to parse walking trials into gait cycles, then data were resampled to 100 data points per cycle. A total of 15 gait cycles, extracted from the middle 20 feet of the walkway to avoid acceleration/deceleration effects, were analyzed for each participant.

### 2.4. Statistical Analysis

#### 2.4.1. Participant Summary

All 15 gait cycles were averaged for each participant to create 10 participant summary curves. Standard deviation across all gait cycles was computed for each participant to describe intra-subject variability. In addition to visual inspection for similar curve shapes, coefficients of multiple correlation (CMCs) [22] were calculated to quantify the agreement of OMC and IMU outputs within each participant’s own walking. CMC values above 0.75 were interpreted as good (0.75–0.84), very good (0.85–0.94), or excellent (0.95–1.00) agreement [22]. Complex CMC values, resulting from taking the square root of a negative number when the intersystem variability exceeded the variability of the motion, are presented as “nan” and should be interpreted as negligible agreement [22,23].

#### 2.4.2. System Summary

All 150 gait cycles were averaged for each system to summarize each system’s performance for each motion. OMC and IMU curves were visually compared, and spread was visualized with standard deviations across all cycles within each motion.

#### 2.4.3. System Differences

The difference between OMC and IMU data at each timepoint for each gait cycle was calculated then averaged across all 150 gait cycles to produce a single difference curve for each motion. An average of the standard deviations of the differences across all gait cycles was determined within each motion to describe variability in system differences across the gait cycle.

#### 2.4.4. Clinical Applications

Kinematics at time of maximum extension or flexion and heel strike have been shown to differentiate disordered from healthy gait [24,25]. Therefore, detecting kinematics similarly with both measurements systems at these timepoints is essential for the systems to be useful alternatives to one another for gait analysis in clinical settings. Sagittal plane kinematics were compared at these critical times during the gait cycle using interclass correlation coefficients estimated using bivariate mixed-effects models in SAS Version 9.4 (SAS Institute Inc., Cary, NC, USA) [26,27]. Interclass correlations are preferred over traditional Pearson product-moment correlation coefficients as the model-based procedure accounts for the hierarchical structure of the data.

Bland–Altman plots were also created to assess bias in and acceptability of the IMU motion capture system as a replacement for OMC. The difference between system measurements (y-axis) were plotted against the averaged value of the two system measurements (x-axis) [28]. Bias for each motion was assessed by comparing the overall average of y-axis data, known as the line of equality, to zero [29]. Measurement agreement was considered acceptable if data fell within ±2.5° from the line of equality [30]. A threshold of 5° was used throughout the analysis as a clinically meaningful difference.

## 3. Results

Ten middle-aged adults with obesity (five female, five male) consented to participate in the study. Participants had an average (±standard deviation) age of 52.8 ± 7.6 years (range: 41–61) and an average BMI of 36.4 ± 3.8 kg/m^2^ (range: 30.9–42.0). Nine participants were White, and one participant was Black/African American.

### 3.1. Participant Summary

**Figure 2:**
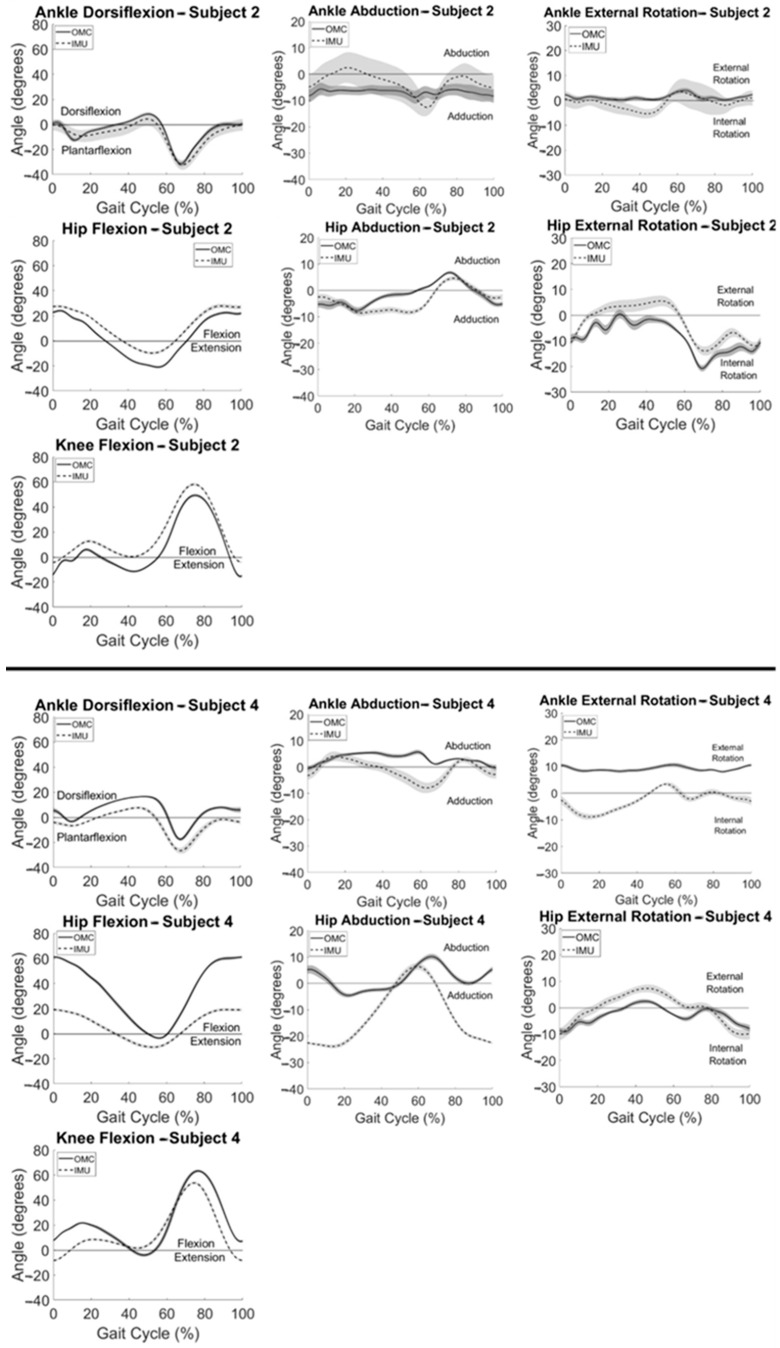
Summary IMU (dashed) and OMC (solid) kinematics vs. % gait cycle for a typical participant with good (**top**) and a typical participant with poor (**bottom**) agreement between systems with one standard deviation in each direction shown in the shaded areas.

Participant summary kinematic curves are shown in Figure 2 for typical subjects with good (Figure 2 (top)) and poor (Figure 2 (bottom)) agreement between systems. Subject 2 (Figure 2 (top)) had the best agreement between systems observed visually and with the largest CMC values for knee flexion, followed by hip flexion, ankle dorsiflexion, hip rotation, and hip abduction (Table 1). Similarly, Subject 4 (Figure 2 (bottom)) had the best agreement between systems observed with knee flexion followed by hip rotation and had poor to no agreement between systems for all other motions (Table 1). Knee flexion showed the greatest agreement between systems for all participants. Within subjects, the largest CMC values at the hip and ankle were observed in the sagittal plane. Some axis-mixing was observed visually in the IMU signal at the ankle, with peaks occurring in the frontal and transverse plane signals corresponding temporally to peaks observed in the sagittal plane.

### 3.2. System Summary

**Figure 3:**
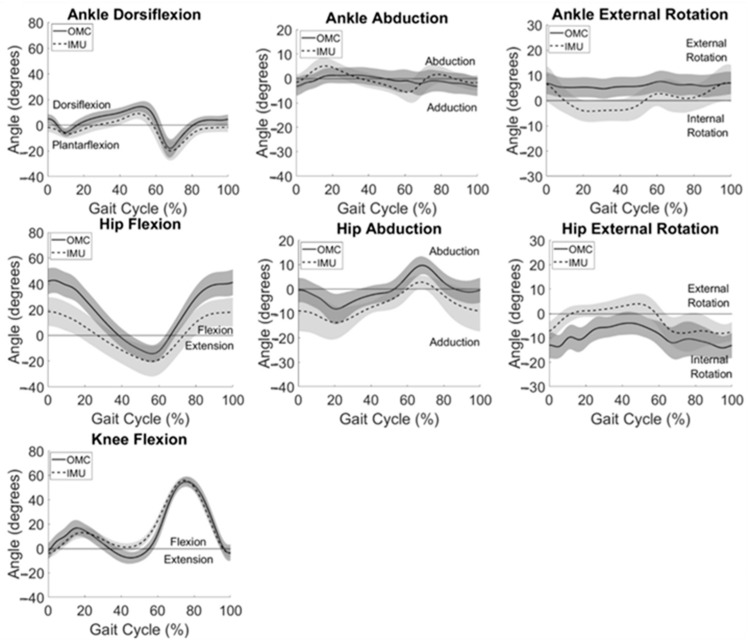
IMU (dashed) and OMC (solid) data averaged across the group for all motions analyzed. One standard deviation in each direction is shown with the shaded areas.

System summary kinematics are presented in Figure 3, averaged across all participants for all gait cycles. Consistent with participant-level observations, system summary curves revealed the greatest agreement between IMU and OMC in the sagittal plane, especially at the knee and ankle. Visual inspection of Figure 3 also reveals similar curve shapes but different measured angle magnitudes for hip frontal and transverse plane movements. Larger standard deviations, shown by wider shaded areas around the mean IMU-measured data when compared to OMC-measured kinematics, can be observed at the hip in all three planes.

### 3.3. System Differences

**Figure 4:**
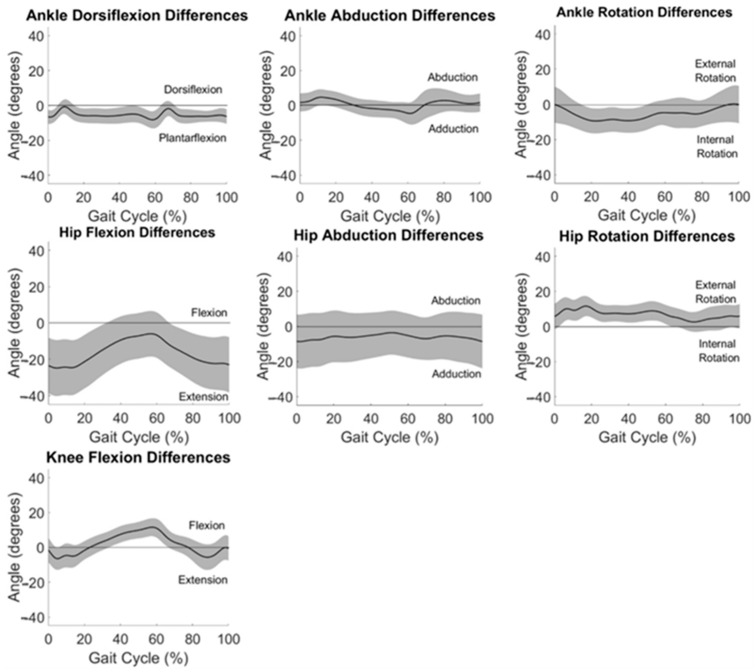
Differences between OMC and IMU data averaged across all participants for all motions analyzed. One standard deviation in each direction is shown in the shaded areas.

Average differences between measurement systems across all participants are displayed in Figure 4. Differences between systems in the frontal and transverse planes are at or near zero degrees throughout the gait cycle for the ankle and hip.

### 3.4. Clinical Application

Sagittal plane angles at critical timepoints during gait were compared between systems with interclass correlation coefficients (Table 2). Ankle dorsiflexion had modest association at heel strike (ρ = 0.38) and good association at minimum angle (ρ = 0.83). All other associations were poor (ρ < 0.20) or even negative.

**Figure 5:**
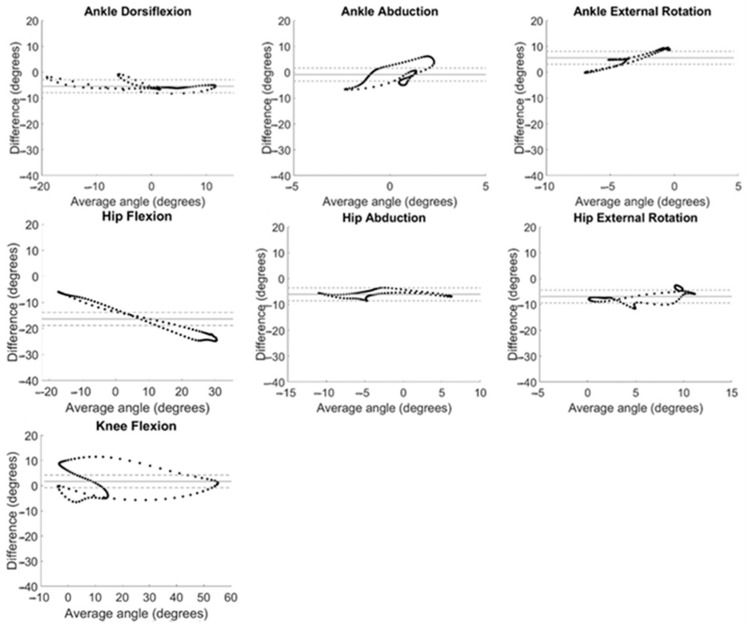
Bland–Altman plots for all kinematic motions analyzed. System differences (OMC − IMU) are plotted against the average of the angles calculated with both systems ((OMC + IMU)/2) at every timepoint during the gait cycle, averaged across all participants. Bias is evaluated by comparing the solid horizontal line (line of equality) to 0°. Agreement is considered acceptable if data fall within a 5° window of the line of equality (dashed lines).

System measurement differences relative to average angle measured at each time point in the gait cycle are displayed as Bland–Altman plots in Figure 5. The horizontal line of equality was near zero, indicating little to no bias for ankle abduction (0.49°) and knee flexion (1.71°), but fell farther from zero for ankle dorsiflexion (5.40°), ankle external rotation (−5.57°), hip abduction (6.07°), hip external rotation (6.92°), and hip flexion (−16.37°). Data fell within a 5° window of measurement acceptability around the line of equality only for hip abduction, though ankle dorsiflexion, ankle abduction, ankle external rotation, and hip external rotation had large ranges of average values in the acceptability window (Figure 5).

## 4. Discussion

The overall objective of this analysis was to evaluate the validity of IMU-based motion capture as a clinically accessible alternative to traditional OMC for assessing gait kinematics in adults with obesity. Sagittal plane kinematics showed the best agreement between systems, especially at the knee. However, not all participants had good agreement between systems at the hip and ankle. Variability in IMU measurements varied across gait for certain motions. Further, only ankle dorsiflexion angles at clinically important times during gait were even modestly associated. Variability in system agreement across participants and poor association between system measurements at timepoints of clinical interest raises concern for the usefulness of IMU-based motion capture for clinical analysis and gait dysfunction diagnosis in this population.

IMU and OMC kinematics were most similar for participants in the sagittal plane, especially with knee flexion. This is consistent with previous work in normal-weight adults which found greatest similarity between an IMU and OMC system in the sagittal plane [31,32]. The systems were most dissimilar at the ankle, where axis mixing in the IMU signal affected kinematic outputs in the transverse and frontal planes. Fewer than half of participants had good agreement for hip abduction and hip external rotation angles, while none had good agreement in the off-sagittal planes at the ankle. Sagittal plane knee motion is largely planar, whereas ankle motion occurs around oblique axes, likely making it difficult for a single IMU on the foot to accurately isolate planar ankle motions relative to the shank. Defining functional axes for ankle kinematics has been shown to be problematic and inconsistent [33,34,35]. The results of this work support the exclusion of off-sagittal plane ankle motion in IMU-based analyses, as has been carried out before [31], due to poor agreement. Frontal plane knee motion has been linked with gait deviations specific to adults with obesity and is strongly related to osteoarthritis development [36,37]. Unfortunately, off-sagittal plane knee motions are not captured with the IMU system when it is used with the recommended software settings, prohibiting this analysis in the present study. However, a similar issue in accurately detecting complex, non-planar motion, as was observed at the ankle, is anticipated for off-sagittal knee kinematics.

Frontal and sagittal plane motion at the hip had a larger spread in IMU data compared to OMC, especially at the beginning and end of the gait cycle (Figure 3). This was not observed in previous work in normal-weight adults [31,32] and therefore may be attributed to the body habitus of participants. Excess bodyweight concentrated around the abdomen and buttock likely affect variability in pelvis IMU measurements which contribute to hip kinematic calculations.

While systems did not agree across all participants, there was no evidence of a systematic offset. Subject-specific factors like anthropometry and IMU placement could have influenced agreement for individual participants. However, all IMU placements were performed by JR who completed company training and consulted with the IMU company to ensure appropriate placement. Therefore, subject-specific differences observed are likely attributed to calibration procedures. The IMU system uses a static standing pose to calibrate baseline “zero” positions for all joints. Individual body shape or standing posture variation will impact IMU orientation during the calibration process and affect joint angle calculations. Previous work comparing IMUs to OMC in normal-weight adults found a similar phenomenon [32] and the authors encourage future work exploring mean-centering data to look at the relative range of motion or exploring functional calibration procedures with the IMU system to eliminate subject-specific biases.

A bias of greater than 5° between system measurements across the range of angles evaluated during gait was evident in ankle dorsiflexion, ankle rotation, hip abduction, hip rotation, and hip flexion. This is in alignment with findings of Berner et al. [31] who found large systematic biases in ankle dorsiflexion (−5.8°) and hip flexion (−7.9°); however, this group found smaller biases (<5°) for hip rotation and abduction and did not evaluate ankle rotation. Our previous work in normal-weight adults found large biases in ankle rotation (−12.1°) [32]. Of note, a linear relationship can be observed for the Bland–Altman plot for hip flexion (Figure 5) demonstrating that the magnitude of the differences between IMU and OMC increased as the hip flexion angle being measured increased. This provides further evidence of the increased variability in the IMU-measured hip flexion observed at the beginning and end of the gait cycle (Figure 3).

Measurement differences between systems differed by more than 5° from the average value for the majority of the range of angles at knee and hip flexion (Figure 5). Only hip abduction had all data within the window of acceptability for the whole range of angles measured. Some ranges of angles for dorsiflexion, ankle abduction, ankle rotation, and hip rotation were found to be acceptable. This is similar to previous findings in normal-weight adults [32] with hip abduction demonstrating the greatest range of data within the window of acceptability and large ranges of data in the window of acceptability for ankle dorsiflexion and hip rotation.

A primary motivation for implementing IMU-based motion capture, a portable and more affordable option than OMC, into clinical settings is to identify subtle gait deviations which warrant early intervention. Early intervention is particularly important among adults with obesity who are at increased risk of disability development and more rapid functional decline compared to their normal weight counterparts [4,5,6]. Sagittal plane kinematics at heel strike and time of maximum or minimum flexion angle has been shown to discriminate disordered from healthy gait using OMC [24,25]. Unfortunately, hip and knee flexion angles measured with both systems at these clinically meaningful timepoints were poorly associated. This warrants caution when using IMU-based motion capture to clinically assess adults with obesity as clinical decisions based on the literature derived from OMC systems but using IMU measurements may be misdirected. Ankle dorsiflexion measured with the IMU system showed modest or good associations with OMC at clinically meaningful timepoints of interest. However, due to the dissimilarity observed between system measurements for ankle kinematics across participants, this finding carries only minor importance.

A limitation of this study is that motion capture with adults with obesity can be challenging due to the presence of subcutaneous adipose tissue. To account for this, an obesity-specific marker set was used [17], and a trained Physical Therapist (JR) palpated for boney landmarks and confirmed placement of all OMC markers. To mitigate effects of motion artifacts distorting data, OMC marker data were low-pass filtered. Further, the Harrington equation [38] was used to estimate hip joint centers. This equation has been shown to be most accurate across many populations and especially in those with a limited range of hip motion [39], as is expected in adults with obesity. IMU-derived kinematics are performed using proprietary software, so the authors took no additional data processing steps to account for IMU motion artifacts. However, IMUs were attached securely to rigid body segments with adjustable straps to minimize sensor movement during gait trials.

Additionally, it is important to note that generalizations of the results of this study to all adults with obesity may be limited given the sample size, lack of racial diversity, and that adults with obesity can be a heterogenous group. Nonetheless, this sample size is consistent with previous validation studies evaluating similar technologies [40,41,42], and the takeaway message indicating limitations in using IMU motion capture as a replacement for OMC was consistent across all participants and over 150 gait cycles. Therefore, the authors feel that the results of this work can be reliably applied to other middle-aged adults with obesity and BMIs below 45 kg/m^2^. To address selection bias in a middle-aged cohort, data collection visits were scheduled for early mornings and evenings when necessary to accommodate work schedules. Additionally, recruitment through the Pitt Plus Me database of over 300,000 ethnically and racially diverse individuals from Western Pennsylvania was used to promote recruitment of a diverse sample. However, participants in this study were primarily White; future studies should include targeted recruitment of a racially and ethnically diverse sample.

Future work should also explore improved calibration procedures to decrease the effects of body shape and standing postures on IMU-based kinematic outputs. Additionally, more work is needed to improve performance of IMU motion capture in off-sagittal planes as these motions are clinically meaningful in the context of musculoskeletal health and disability development. Until these improvements are made, the authors urge caution when implementing and interpreting kinematics from IMU systems in adults with obesity.

## 5. Conclusions

Wearable sensor-based motion capture allows for biomechanical assessment of gait in clinical settings, aiding in early detection of movement deviations indicating disability development risk. Leveraging this technology for adults with obesity is particularly important because this population is at increased risk for rapid functional decline. The results of this study show that IMU-measured gait kinematics is most similar to the reference standard OMC in the sagittal plane. However, kinematics are poorly associated at clinically meaningful timepoints and large differences between system measurements exist across certain ranges of motion. As such, caution should be used when applying IMU-based motion capture in adults with obesity for clinical assessment. Future work should improve standardization procedures for IMU calibration and explore methods to adjust for obesity-specific considerations in IMU motion capture performance before this technology can be responsibly implemented into clinical settings.

## Figures and Tables

**Table 1:** Coefficient of multiple correlation (CMC) values for all motions within each participant.

	Ankle Dorsiflexion	Ankle Abduction	Ankle Rotation	Hip Flexion	Hip Abduction	Hip Rotation	Knee Flexion
Subject 1	0.59	nan ^#^	nan ^#^	**0.95**	nan ^#^	nan ^#^	**0.99**
Subject 2 ^a^	**0.91**	nan ^#^	nan ^#^	**0.92**	**0.75**	**0.79**	**0.94**
Subject 3	0.65	nan ^#^	0.30	**0.80**	**0.88**	0.34	**0.98**
Subject 4 ^b^	0.71	nan ^#^	nan ^#^	0.33	nan ^#^	**0.75**	**0.89**
Subject 5	**0.98**	nan ^#^	nan ^#^	**0.77**	0.56	**0.78**	**0.96**
Subject 6	**0.95**	nan ^#^	nan ^#^	**0.76**	**0.90**	nan ^#^	**0.92**
Subject 7	**0.97**	0.37	nan ^#^	nan ^#^	nan ^#^	0.50	**0.97**
Subject 8	**0.93**	nan ^#^	nan ^#^	0.64	nan ^#^	**0.87**	**0.93**
Subject 9	0.67	nan ^#^	nan ^#^	**0.94**	0.56	nan ^#^	**0.97**
Subject 10	**0.80**	0.39	nan ^#^	0.50	**0.94**	0.31	**0.99**

^a^ data in Figure 2 (top), ^b^ data in Figure 2 (bottom), ^#^ “nan” indicates that CMC is a complex number and should be interpreted as 0 or negligible. **Bolded** CMC values are ≥0.75, showing good–excellent agreement between measurement systems.

**Table 2:** Interclass correlation coefficients (ρ) at gait cycle time points of interest between IMU and OMC sagittal plane angles.

		Timepoint	
		Heel Strike	Maximum/Minimum
Motion	Ankle Dorsiflexion	0.38	0.83
	Hip Flexion	−0.05	0.15
	Knee Flexion	0.18	0.00

## Data Availability

Deidentified participant data relevant to the results reported in this manuscript, along with the full study protocol, will be available upon request. Data will be made available electronically for 5 years to researchers whose proposed analysis plan is approved by the investigative team.
